# IL-38 and IL-36 Target Autophagy for Regulating Synoviocyte Proliferation, Migration, and Invasion in Rheumatoid Arthritis

**DOI:** 10.1155/2021/7933453

**Published:** 2021-11-20

**Authors:** Zhe Hao, Yi Liu

**Affiliations:** Department of Rheumatology and Immunology, West China Hospital, Sichuan University, Chengdu, 610041 Sichuan, China

## Abstract

Rheumatoid arthritis (RA) is an autoimmune disease leading to severe joint damage and disability. Fibroblast-like synoviocytes (FLSs) mostly contribute to the joint inflammation and destruction in RA through distinct mechanisms. However, little is known about newly discovered interleukin- (IL-) 36 and IL-38 involving in the pathology of RA. Here, we assessed the effect of IL-36 and IL-38 on RA-FLS function using IL-36 and IL-38 overexpression plasmids. We found that IL-36 inhibited synoviocytes proliferation while IL-38 showed an opposite influence. Furthermore, IL-36 and IL-38 significantly sequestered or accelerated RA-FLS migration and invasion capacity, respectively. Mechanically, IL-36 and IL-38 targeted autophagy for RA-FLS modulation. Using autophagy inhibitor 3-MA and inducer compound rapamycin, we found that autophagy negatively regulated the survival, migration, and invasion of synovial cells. Based on these results, IL-38 in combination with autophagy inhibitor 3-MA treatment demonstrated the strongest blockage of the above three activities of RA-FLS, and IL-38 overexpression reversed rapamycin-inhibited cell proliferation, migration, and invasion. Moreover, injection of IL-36 can improve the symptoms of RA in a rat model of RA. Taken together, we conclude that IL-38 and IL-36 target autophagy for regulating synoviocyte proliferation, migration, and invasion in RA.

## 1. Introduction

Rheumatoid arthritis (RA) is a chronic, inflammatory, systemic, autoimmune arthritides characterized by immunoglobulin G and citrullinated protein-specific autoantibodies induced synovial inflammation and hyperplasia, ultimately leading to cartilage damage and bone destruction [1]. It is related to systemic disorders such as cardiovascular, pulmonary, and skeletal dysfunction, among which accompanied cardiovascular disease ascribes to the most common cause of mortality of RA patients [2]. Epidemiological researches have reported that RA has a fairly constant global prevalence with the rage of 0.5-1.0% in white population [3]. The cause of the disease is complex and is associated with interplays of risking factors like genetics, female sex, and environmental factors. Some biomarkers have been found to be related with RA [4, 5]. During the progress of RA, the resident fibroblast-like synoviocytes (FLSs) assume a semiautonomous and aggressive phenotype, constituting the fundamental mechanism for RA pathogenesis [6]. RA-FLS can proliferate in an anchorage-independent manner without contact inhibition. The increasing number of FLS thus contributes to the transformation of synovial lining into invasive pannus, which can migrate between joints and is directly responsible for cartilage and bone damage [7]. In addition, activated RA-FLS can produce a range of inflammatory cytokines, chemokines, and proangiogenic factors, facilitating the recruitment and proliferation of immune cells such as T cells and B cells, to maintain adaptive immune organization and joint inflammation as well as joint angiogenesis [8]. Therefore, instead of focusing on altering proinflammation cytokines and the immune signaling, targeting RA-FLS to deactivate its autonomous and aggressive phenotypes can be promising strategies for RA therapy.

Interleukin- (IL-) 38 and IL-36 belong to IL-1 cytokine family, which can be divided into three subfamilies as IL-1, IL-18, and IL-36 subfamily by precursor protein length, and play crucial rules in innate and adaptive immunity [9]. Recent studies have suggested both proinflammation and anti-inflammation properties of IL-38, depending on the protein concentration, existing form, posttranslational modification, or cellular environmental context. However, in most cases, IL-38 acts as an antagonist of receptors IL-1R1, IL-36R, or IL-1RAPL1 or recruits inhibitory coreceptor like single Ig IL-1-related receptor (SIGIRR) to suppress proinflammation responses. By inhibiting their binding with specific ligands like IL-1*β*, IL-33, or IL-36, IL-38 sequesters downstream NF-*κ*B, AP1, and JNK signaling pathways [10], therefore, mechanically regulating the progression of numerous disease comprising cancer, myocardial infarction, and autoimmune diseases such as RA [11]. Indeed, emerging studies have indicated that IL-38 is involved in RA pathology. First of all, IL-38 expression was found significantly improved in the serum, synovial tissue, and synovial fluid in RA patients and was associated with production of inflammation cytokines like IL-1*β*, IL-6, or IL-1Ra [12]. Gene polymorphism of IL-38 was also related to the occurrence of RA [13]. Moreover, IL-38 administration was found to alleviate RA severity in both collage-induced and K/BxN-induced RA mouse model through inhibiting proinflammation responses [14]. However, the function of IL-38 in RA besides immune regulation has not been well described yet.

Unlike IL-38, IL-36 exhibit proinflammation characteristic through heterodimeric receptor complex IL-36R and IL-1RAcP. Upon binding to the receptors, Myd88 is recruited and phosphorylates IRAKs, followed by TRAF6 conjunction and ubiquitination, and ultimately activates MAPK and NF-*κ*B signaling pathway, leading to the production of IFNs, inflammation cytokines, and chemokines like IFN-*γ*, IL-8, or CXCL1 [15]. It has been reported that IL-36 expression is related to many diseases such as psoriasis, arthritis, obesity, and other chronic diseases [16]. IL-36 and IL-36R were both found elevated in the synovium in RA patients and RA-inducing animal models, with the ability to activate IL-6 and IL-8 secretion in RA-FLS [17, 18], while the molecular mechanism of IL-36 in RA pathology remains to be studied.

Autophagy is a highly conserved endogenous self-degradation process for organelle, proteins, or intracellular pathogens [19], thus maintaining cellular homeostasis and deciding cell fate. Autophagy is a dynamic and multiple-step cellular process, involving initiation, phagophore nucleation and elongation, autophagosome maturation, and autolysosome fusion and degradation [20]. Emerging evidence has identified the importance of autophagy in rheumatic disease, such as systemic lupus erythematosus, rheumatoid arthritis, or osteoarthritis [21, 22]. In RA, many different types of cells present disorder of autophagy, including FLSs, activated T cells, and osteoblast, all of which largely contribute to the pathogenesis of RA [23–25]. Dysfunction of autophagy in RA-FLS leads to hyperplasia of synovial tissue, and downregulated autophagy in cartilage cells is responsible for cell death and cartilage destruction. Mechanically, autophagy results in apoptosis resistance in synovium, T cells, and osteoclasts, promoting their proliferation and survival, therefore, exacerbating RA progression. The function of autophagy in other molecular mechanisms of RA is complex and not well established.

Therefore, we hypothesized that IL-38 and IL-36*α* may regulate the disease progression of RA, and autophagy may be involved in the process. In this study, we sought to validate the crosstalk of inflammation cytokines IL-36/IL38 and autophagy in determining the process of RA pathology, trying to provide some new insights in RA regulation. The therapeutic effect of IL-36 for RA was also primarily investigated.

## 2. Materials and Methods

### 2.1. Cell Culture and Stimulation

SW982 cell line was purchased from the Cell Bank of Type Culture Collection of Chinese Academy of Sciences and was cultured in Leibovitz's L-15 Medium (Solarbio, Beijing, China) containing 10% fetal bovine serum (Thermo Fisher, Waltham, MA, USA), 100 U/mL, penicillin and 100 mg/mL streptomycin at 37°C in 5% CO2, respectively. For autophagy inhibition or initiation, cells were treated with 10 mmol/L of 3-MA or rapamycin (10 nmol/L; Sigma Aldrich, St. Louis, MO, USA) for 24 hours, after transfection with recombinant plasmid.

### 2.2. Plasmid Construction and Transient Transfection

IL-38 and IL-36 gene sequence information were obtained from NCBI gene database and the target IL-38 or IL-36 DNA fragment was directly synthesized with NheI and KpnI restriction enzyme cutting site added to N-terminal or C-terminal, respectively. Target gene was inserted ahead of EGFP gene sequence into pcDNA3.1-EGFP vector for fusion protein expression. After double-enzyme digesting, DNA ligase, transduction, and bacterial colony amplification, plasmids were extracted for NheI and KpnI digestion and sequencing validation. Plasmid DNA was transfected into cells using lipofectamine 2000 transfection reagent (Thermo Fisher) according to manufacturer's instruction.

### 2.3. RNA Extraction and Quantitative Real-Time PCR

Cells were harvested 24 hours after transfection with indicated plasmids and lysed with Trizol reagent (Thermo Fisher). Total RNA was isolated according to manufacturer's procedure. Briefly, after lysing in 1 volume of Trizol for 5 min, 1/5 volume of chloroform was added for homogenization vigorously and incubated for 3 min. After centrifuging at 12000 g, 4°C for 15 min, the upper aqueous phase was gathered, and 1/2 volume of isopropanol was added for incubation at room temperature for 10 min, followed by centrifuging at 12000 g, 4°C for 15 min. The RNA pellet was washed with 1 mL 75% ethanol, centrifuged at 12000 g, 4°C for 5 min. After air dried, RNA was dissolved in RNase-free water and stored at -70°C until use.

For quantitative real-time PCR analysis, PrimeScript II Reverse Transcriptase (Takara, Japan) was used for first-strand cDNA synthesis according to manufacturer's protocol. The cDNA was amplified, and gene expression was determined with Bio-Rad CFX connect detection system using iTaq Universal SYBR Green Supermix (Bio-Rad, Hercules, CA, USA). PCR amplification was performed as follows: 95°C for 5 min followed by 40 cycles consisting of 95°C for 15 s, 55°C for 15 s, and 72°C for 30 s. mRNA transcript levels were normalized to GAPDH. The following gene-specific primer sequences were used: 5′-GGGAAACTGTGGCGTGAT-3′ (sense) and 5′-GAGTGGGTGTCGCTGTTGA-3′ (antisense) for GAPDH; 5′-ACCAACCCGAGCCTGTGA-3′ (sense) and 5′-CCCAGTTCTTGGGTAAGGATG-3′ (antisense) for IL-36; 5′-GACAACTGCTGTGCAGAGAAG-3′ (sense) and 5′-GGCCTCTTCACCACCTTTGT-3′ (antisense) for IL-38.

### 2.4. Western Blot

Cells were lysed in RIPA buffer with PMSF (Solarbio, Wuhan, China), and extracted-protein concentration was determined using Bio-Rad Protein Assay (Bio-Rad). Western blot analysis was performed with indicated antibodies in accordance with the Western blotting protocol provided by Cell Signaling Technology with modification. Specific antibody against IL-38 was purchased from Abcam (Cambridge, MA, USA). Primary antibodies against GAPDH, IL-36*α* and LC3, and HRP-conjugated goat-anti-rabbit IgG secondary antibody were obtained from Bioswamp (Wuhan, China). Briefly, protein samples were loaded onto SDS-PAGE gel and electrotransfered to PVDF membrane, then incubated the membrane in blocking buffer for 1 h at room temperature followed by primary antibody (1 : 1000 dilution) incubation with gentle agitation overnight at 4°C. The next day, the membrane was incubated with species appropriate for HRP-conjugated secondary antibody in blocking buffer (1 : 20000 dilution) with gentle agitation for one hour at room temperature. The proteins were detected with Clarity Western ECL Substrate (Bio-Rad) for digital imaging using Tannon-5200 (Tannon, Shanghai, China).

### 2.5. Immunofluorescence Assay

SW982 cells were plated in 35 mm confocal dishes (Ibidi, Lochhamer, Germany). After treatment, cells were fixed with 4% paraformaldehyde in PBS pH 7.4 for 10 min at room temperature. Then, permeabilization was performed by incubating the samples for 10 min at 4°C with PBS containing 0.1–0.25% Triton X-100. Samples were then blocked in PBS containing 5% normal serum and 0.3% Triton X-100 for 1 hour at room temperature, followed by incubation with diluted primary antibody in PBS containing 1% BSA and 0.3% Triton™ X-100 overnight at 4°C. Next day, samples were incubated with Alexa Fluor 594-conjugated goat anti-rabbit secondary antibody (Bioswamp, Wuhan, China) for one or two hours in the dark before nuclear staining with DAPI for 5 min at room temperature. Dishes were stored at 4°C protected from light for long-term storage. Cells were finally imaged using inverted immunofluorescent microscope (DMIL LED, Leica, Wetzlar, Germany).

### 2.6. EdU Proliferation Assay

Cell Light Edu Apollo 567 In Vitro Imaging Kit was supplied by Guangzhou RiboBio (China, Guangzhou) and used for determining the proliferation of SW982 cells according to manufacturer's instructions. After experimentation, 100 *μ*L, 50 *μ*M EdU was added into the cell culture medium for incubation for 2 hours. Fixation was performed at room temperature for 15 min using 4% paraformaldehyde diluted in PBS, followed by permeabilization with 0.5% Triton X-100 and incubated 20 min at room temperature. Fluorescent EdU detection was performed after EdU staining with Apollo567 and nuclear staining with Hoechst 33258. Images were collected using immunofluorescent microscope (DMIL LED, Leica).

### 2.7. Wound Healing Assay

Wound healing assays were performed to determine the migration ability of SW982 synovial cells after different treatments. Reference lines were marked to guarantee the same area of image acquisition. Cells were seeded into 24-well plates and grew to 80% confluence for transfection or stimulation. 24 hours later, a linear wound was created in the cellular monolayer with a same 200 *μ*L pipette tip in each well. After scratch, gently wash cell monolayer to remove detached cells. Cells were continually cultured at 37°C with 5% CO2, and images of the wound were captured at 0, 24, or 48 hours upon the wound creation using a phase contrast inverted microscope with appropriate magnification.

### 2.8. Transwell Cell Invasion Assay

The cell invasion assays were performed using Corning BioCoat Matrigel invasion chamber (Corning, NY, USA), with a pore size of 8 *μ*m polyethylene terephthalate membrane precoated with Matrigel. SW982 cells transfected with indicated plasmids or treated with correspondent chemical reagents were resuspended in serum free medium and were seeded in the upper well at the concentration of 104/mL.

L-15 media containing 20% FBS was added to the lower chamber as the chemoattractant. Cells were incubated for 48 hours, and noninvaded cells were gently removed; the remaining cells downside of the membrane surface were stained with 0.1% crystal violet at room temperature for 5 min. Cells were imaged with Inverted Laboratory Microscope Leica DM IL LED and analyzed from randomly selected different fields.

### 2.9. Animal Model of RA

Fifteen specific-pathogen-free (SPF) male SD rats (weighing 180-220 g) were purchased from Hubei Animal Experiment Center. Rats were maintained under SPF conditions with a room temperature of 22-26°C and humidity of 50-60% under a 12-hr light/dark cycle with access to food and water ad libitum. All animal procedures were approved by the Institutional Animal Care and Use Committee of the local institution.

Rats were randomly divided into three groups: control, model, and treatment groups (*n* = 5 per group). Rats in the model and treatment groups were injected with 50 *μ*L of type II collagen (2 mg/mL) combined with incomplete Freund's adjuvant in the articular cavity of hind ankle joint at the first and 7^th^ day. Rats in the control group were injected with the same volume of incomplete Freund's adjuvant. Rats in the treatment group were injected with recombinant IL-36 from the first day to the 10^th^ day. All rats were sacrificed until the 28^th^ day. Before sacrifice, the body weight and the swelling and color of the feet and joints were recorded, and the length and thickness of the ankles and feet were measured and compared. After the rats were sacrificed, the ankle joint synovium was collected for hematoxylin-eosin (HE) staining to observe the inflammation and infiltration.

### 2.10. HE Staining

Collected ankle joint synovium were fixed in 10% formaldehyde for more than 24 hr. The samples were dehydrated in alcohol, embedded in paraffin, and cut into 3 *μ*m sections. Subsequently, the sections were deparaffinized and rehydrated, then subjected to HE staining following a standard protocol. Images were captured under a microscope (DMIL LED, Leica, Wetzlar, Germany).

### 2.11. Statistical Analysis

The data are expressed as the means ± SD and are representative of three independent experiments. Statistical differences between the experimental groups were calculated using GraphPad Prism software. The means obtained for the different groups were compared by one-way ANOVA followed by the Tukey post hoc test. A value of *P* < 0.05 was considered statistically significant.

## 3. Results

### 3.1. IL-38 and IL-36 Regulate Synoviocyte Proliferation, Migration, and Invasion

**Figure 1:**
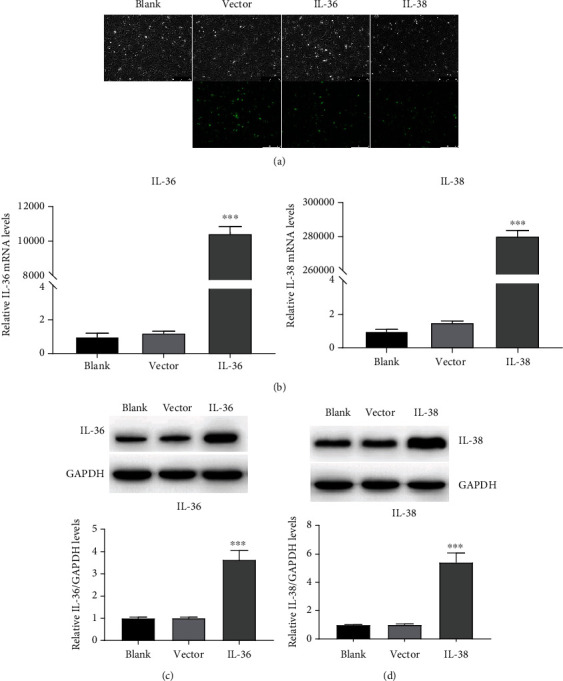
Identification of IL-38 and IL-36 overexpression construct. (a) SW982 cells were transfected with EGFP fusion IL-36 or IL-38 encoding plasmids or empty vector. Cells were examined under microscopy (100x) for EGFP expression. Representative images are presented from three biological replicates. (b) SW982 cells were transfected with empty vector or IL-36 (left panel) or IL-38 (right panel) or left untransfected, followed by total RNA isolation 24 hours posttransfection and subjected to qRT-PCR analysis. (c) SW982 cells were transfected with the indicated plasmids for 24 hours followed by Western blot detection for IL-36 or IL-38 expression; GAPDH was used as control. Data represent the mean from three independent experiments with SD. ^∗∗∗^*P* < 0.001 compared to the control group.

First, we want to determine the effect of IL-36 and IL-38 on the proliferation of FLS in RA pathology. The expression of IL-38 and IL-36 overexpression construct was first assessed. As shown in Figure 1(a), both IL-36 and IL-38 encoding plasmid successfully expressed plenty of EGFP fusion protein validated by microscope examination. mRNA and protein expression of IL-36 and IL-38 were confirmed with RT-PCR and Western blot (Figures 1(b)–1(d)).

**Figure 2:**
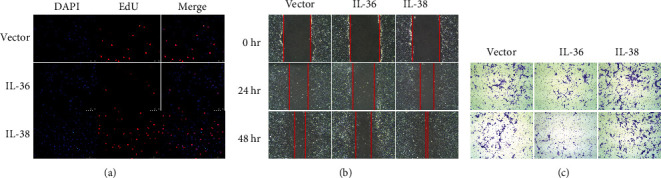
IL-38 and IL-36 regulate synoviocyte proliferation, migration, and invasion. (a) SW982 cells transfected with indicated plasmids for 24 hours were incubated with EdU followed by fixation and permeabilization and then subjected to EdU staining with Apollo567 and nuclear staining with Hoechst 33258. Representative micrographs (200x) from three independent experiments are shown. (b) SW982 cells were transfected with the indicated constructs for 24 hours followed by scratching to form a linear wound with pipette tip. Cells were then cultured for indicated hours, and images of the wound were captured using a phase contrast inverted microscope with 100x magnification. (c) SW982 cells with IL-36 or IL-38 or empty vector transfection for 24 hours were digested by trypsin and resuspended and placed into upside of the transwell. Cells were cultured for 48 hours and were determined by crystal violet staining and microscopy (100x). Representative images are presented from at least three biological replicates.

EdU cell proliferation assay was performed to evaluate the proliferation of SW982 cells transfected with IL-36 or IL-38 expression plasmids. The results showed that compared with the control, IL-36 overexpression reduced the cell proliferation, whereas IL-38 overexpression increased the cell proliferation (Figure 2(a)). Then, the effect of IL-36 and IL-38 on the migration and invasion of SW982 cells was investigated. As shown in Figure 2(b), the wound widths in IL-38 overexpressed cells were dramatically narrowed at both 24 hr and 48 hr. However, cells that migrated toward the scratch were attenuated by IL-36. Simultaneously, the number of cells penetrated through Matrigel-coated transwell membranes increased significantly in the presence of IL-38 while the cells infiltrated into the other side of the monolayer filter were suppressed by IL-36 overexpression. These data demonstrated that IL-38 facilitated while IL-36 impaired the migration and invasion capacity of SW982 cells. Taken together, our results suggested that IL-36 and IL-38 regulated proliferation, migration, and invasion of the synoviocytes in an opposite manner.

### 3.2. IL-38 and IL-36 Modulate Autophagy

**Figure 3:**
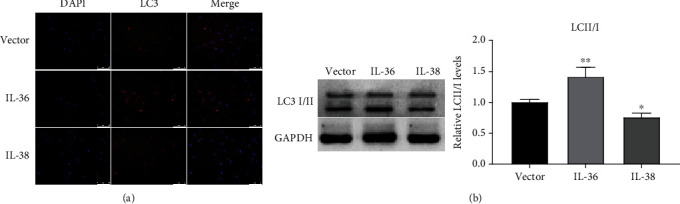
IL-38 and IL-36 modulate autophagy. (a) SW982 cells were transfected with IL-36 or IL-38 overexpression construct or empty vector for 24 hours. Cells were then subjected to immunofluorescent staining using LC3 antibody, and nucleus were labeled with DAPI. Representative micrographs (100x) from three dependent experiments were shown. (b) SW982 cells with the indicated plasmids transfection for 24 hours were harvested and lysed in RIPA buffer and subjected to SDS-PAGE and Western blot analysis. The endogenous LC3I/II turnover was assessed using LC3 specific antibody. GAPDH was detected as control. The data are representative of three independent experiments. ^∗^*P* < 0.05, ^∗∗^*P* < 0.01 compared to the vector group.

Next, the effect of IL-36 and IL-38 on autophagy in SW982 cells was investigated. LC3-I is diffuse cytosolic distributed and LC3-II puncta is focus distributed, which can be indicated by the bright intensity and location of the fluorescence. As shown in Figure 3(a), fluorescence was focused and bright in the IL-36 expression group while was diffused in the IL-38 overexpression group. Western blot results in Figure 3(b) showed that IL-36 potentiated conversion of LC3-I into LC3-II while IL-38 suppressed LC3-II accumulation. These data demonstrated that IL-36 accelerated and IL-38 impeded autophagy.

### 3.3. IL-38 Promotes Autophagy-Inhibited Cell Proliferation

**Figure 4:**
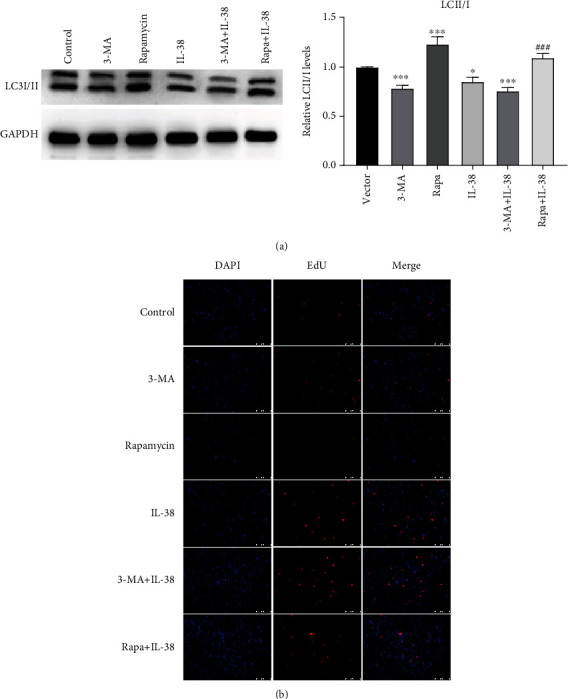
IL-38 promotes autophagy-inhibited cell proliferation. (a) SW982 cells were transfected with IL-38 overexpression plasmids or empty vector and treated with 3-MA or rapamycin or left untreated. Cells were cultured for 24 hours and then collected and lysed for immunoblotting analysis. The endogenous LC3I/II turnover was assessed using LC3 specific antibody. GAPDH was detected as control. ^∗^*P* < 0.05, ^∗∗∗^*P* < 0.001 compared to the vector group; ^###^*P* < 0.001 compared to the Rapa group. (b) SW982 cells were transfected with empty vector or IL-38 overexpression plasmids and treated with 3-MA or rapamycin or left untreated. Cells were cultured for 24 hours before EdU proliferation assay. Representative micrographs (200x) from three independent experiments are shown.

Since IL-38 impeded autophagy, next, we investigated whether IL-38 regulates RA-FLS proliferation via autophagy. SW982 synovial cells were treated with autophagy initiation inhibitor 3-methyladenine (3-MA) or inducer rapamycin (Rapa) with or without IL-38 encoding plasmids transfection. As shown in Figure 4(a), in the untransfected group, LC3-II turnover was alleviated by 3-MA and exacerbated by rapamycin treatment. Immunoblot analysis also indicated that IL-38 inhibited LC3-II formation, and this inhibition was a little aggravated by the addition of 3-MA. Moreover, IL-38 overexpression mitigated rapamycin-induced LC3-II accumulation comparison with only rapamycin-treated group. EdU staining assay showed that inhibition of autophagy by 3-MA promoted SW982 cell proliferation, and by contrast, initiation of autophagy by rapamycin greatly restrained the red fluorescent light intensity (Figure 4(b)). In addition, IL-38 overexpression significantly enhanced cell proliferation in both untreated or 3-MA or rapamycin-treated groups. Combination treatment of IL-38 and 3-MA exhibited the strongest ability to accelerate cell proliferation, and IL-38 overexpression reversed the rapamycin-mediated blockage of cell proliferation. In conclusion, these data demonstrated that IL-38 promoted autophagy-inhibited synovial cell proliferation.

### 3.4. IL-38 Facilitates Synoviocyte Migration and Invasion via Autophagy

**Figure 5:**
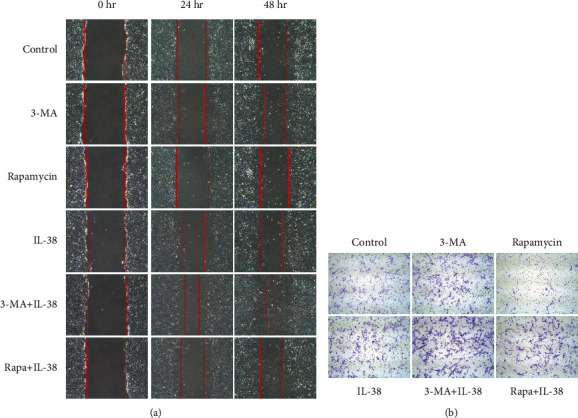
IL-38 facilitates synoviocyte migration and invasion via autophagy. (a) SW982 cells were transfected with IL-38 overexpression plasmids or empty vector and treated with 3-MA or rapamycin or left untreated. Cells were cultured for 12 hours followed by wound healing assay. Images of the wound were captured at 0, 24, and 48 hours after the wound was created. Representative micrographs (100x) from three independent experiments are shown. (b) S SW982 cells were transfected with IL-38 overexpression plasmids or empty vector and treated with 3-MA or rapamycin or left untreated. Cells were cultured for 24 hours and then digested by trypsin, resuspended, and seeded into upside well of the transwell system. After cultured for 24 hours, cells were subjected to crystal violet staining and microscopy examination. Three separate experiments were performed with consistent results, and represented images are shown.

Then, we investigated how autophagy regulated synovial cell migration and invasion, as well as the role of L-38 in these processes. Wound healing analysis showed that 3-MA treatment enhanced the migration ability of SW982 cells at both 24 hr and 48 hr, and autophagy initiated by rapamycin slowed down cell migration (Figure 5(a)). In addition, we found that IL-38 overexpression group displayed similar narrow wound width in comparison with 3-MA group. In line with this, IL-38 and 3-MA treatment together maximumly improved cell wound healing speed, while IL-38 restored the cell healing capacity diminished by rapamycin. These data collectively suggested that IL-38 promoted synoviocyte migration, which was inhibited by autophagy.

Next, we evaluated the influence of IL-38 and autophagy on cell invasion using transwell assay. As shown in Figure 5(b), 3-MA treatment led to increasing SW982 cells translocated from the coated filter to the downside serum containing culture medium. Rapamycin addition alleviated the number of cells stained with crystal violet, suggesting impaired invasion ability. After IL-38 transfection, more cells managed to move across the transwell membrane compared to the control group. In line with the aforementioned results, IL-38 overexpression accelerated 3-MA-induced cell invasion and reversed the rapamycin downregulated number of transmembrane cells. Taken together, these findings demonstrated that IL-38 facilitates synoviocyte migration and invasion via autophagy.

### 3.5. IL-36 Treatment Improve the Symptoms of RA in a Rat Model

**Figure 6:**
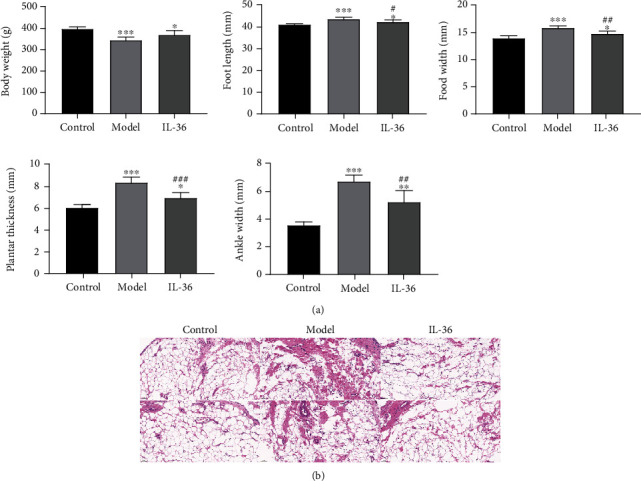
IL-36 treatment improve the symptoms of RA in a rat model. A rat model of RA was developed by injecting type II collagen combined with incomplete Freund's adjuvant to the articular cavity of hind ankle joint of the rat at the first and 7^th^ day. Rats in the treatment group were injected with recombinant IL-36 from the first day to 10th day. All rats were sacrificed until the 28^th^ day. (a) The body weight and the length and thickness of the ankles and feet were measured and compared. ^∗^*P* < 0.05, ^∗∗^*P* < 0.01, ^∗∗∗^*P* < 0.001 compared to the vector group; ^#^*P* < 0.05, ^##^*P* < 0.01,^###^*P* < 0.001 compared to the model group. (b) HE staining of the ankle joint synovium to show the inflammation and infiltration.

Since IL-36 showed protective effect against synoviocytes, we next explored the therapeutic effect of IL-36*α* in the rat model. The morphological observation results showed that compared with the control group, the weight of the rats in the model and treatment groups was significantly reduced, and the weight of the rats in the treatment group was higher than that of the model group without significant difference (Figure 6(a)). The foot length, width, plantar thickness, and ankle width of rats in the model and treatment groups were significantly larger than those of rats in the control group, but these indicators of rats in the treatment group were significantly lower than those of the model group (Figure 6(a)). The HE staining results showed that the cells in the control rats were neatly arranged without inflammatory cell infiltration, but cells in the model rats had severe inflammatory cell infiltration, while cells in the treatment rat had almost no inflammatory cell infiltration, which was comparable to that in the control group (Figure 6(b)). These data suggest that IL-36 treatment can improve the redness and swelling of the paws, relieve the symptoms of paw arthritis, and inhibit the inflammation in the joint synovium.

## 4. Discussion

In this study, we found that IL-38 enhanced synovial cell proliferation, migration, and invasion while IL-36 exhibited contrary function. IL-36 accelerated and IL-38 impeded synovial cell autophagy, which may contribute to RA pathology. Further experiments showed that IL-38 promoted autophagy-restrained synovial cell proliferation, migration, and invasion. Moreover, injection of IL-36 can improve the symptoms of RA in a rat model of RA. Our data together suggested crucial roles of IL-38 and IL-36 for determining RA-FLS fate and ultimately regulating joint damages.

RA is a chronic inflammatory disease with the main pathological feature of proliferative synovial lining tissue caused by hyperplasia of FLS [6]. RA is also characterized as cartilage and bone destruction caused by sybovium transformation into the invasive tissue and aggressive phenotypes of FLSs such as migration and invasion [26]. Both IL-38 and IL-36 were found to be elevated in RA patients [12, 13, 17, 18]. The present study found that IL-38 enhanced while IL-36 inhibited synovial cell proliferation, migration, and invasion. IL-38 can bind with IL-36R and function as an antagonist to IL-36, thus interrupting IL-36 binding to heterodimeric receptor complex IL-36R/IL-1RAcP [27]. IL-36 binds to the IL-36R leading to nuclear factor kappa B/mitogen-activated protein kinase-mediated cytokine release [15]. Recently, a study reported that IL-36R may mediate the crosstalk between plasma cells and FLS [28]. Therefore, by competing IL-36R, elevated IL-38 and IL-36 together promoted FLS proliferation and migration, thus contributing to the pathogenesis of RA. Moreover, our preliminary study in rats showed that IL-36 treatment can relieve the symptoms of paw arthritis and inhibit the inflammation in the joint synovium of RA rats, indicating its possible clinical application.

Autophagy is a fundamental intracellular degradation process with multiple roles in immunity, thus maintaining cellular homeostasis and deciding cell fate [19]. Recent studies have revealed the involvement of autophagy in RA pathogenesis including elevated autophagy in RA-FLS for apoptosis resistance [29, 30]. Dysfunction of autophagy in RA-FLS leads to hyperplasia of synovial tissue, and downregulated autophagy in cartilage cells is responsible for cell death and cartilage destruction. The present study found that IL-36 accelerated and IL-38 impeded synovial cell autophagy. For now, there are barely a few references studying the relationship of IL-38 and IL-36 with autophagy. A recent report in 2020 reported that IL-36 activated autophagy to ablating the immune inhibitory of CD4+CD25+ Treg [31], thus aggravating host immune response and the development of sepsis. This is consistent with our conclusion that IL-36 promoted autophagy in RA for synoviocyte hyperplastic activity. No previous work characterized the role of IL-38 in autophagy. In consideration of the resemblance in anti-inflammation, IL-10 was described to suppress autophagy through activating PI3K/Akt/mTOR and JAK/STAT signal pathway. Indeed, de Graaf et al. currently found that IL-38 prevented the induction of trained immunity by inhibition of mTOR signaling [32]. This finding could be one theoretical foundation of our study. Also, IL-38 signaling results in the production of many inflammatory cytokines such as IL-2, IL-6, IL-13, and IL-17, all of which have the ability to participate in the autophagy process. IL-6 signals through STAT3/Bcl-2 to decline the expression of autophagy protein Beclin 1 for autophagy inhibition [33]; IL-17 activates TAB2/3-mediated MAPK pathway to block autophagy progression [34]. Therefore, IL-38 might indirectly target autophagy for RA regulation.

IL-38 and IL-36 affect synovial cell proliferation, migration, and invasion, and they also modulate synovial cell autophagy. Autophagy is reported to modulate cell migration and invasion through different mechanisms [35–37]. Therefore, the effect of IL-38 and autophagy interaction on synovial cell proliferation, migration, and invasion was further studied. IL-36 was not further studied due to its inhibited effects on RA-FLS biological process. Our data showed that IL-38 promoted synovial cell proliferation, migration, and invasion via autophagy. Distinct mechanisms of pathogenesis and progression of RA are positively and negatively related to autophagy. Autophagy contributes to the microorganism elimination or immunological tolerance to prevent RA, whereas autophagy accelerates citrullinated antigen presentation and T cell and B cell activation [38]. Therefore, the modulation of cell events of IL-38 via autophagy may contribute to the immune regulation during RA.

In conclusion, here, we propose a working model of IL-36 and IL-38 regulating FLS function via autophagy, which connects newly discovered IL-36/IL-38 immune regulation with autophagy, providing the possibility of clinical application targeting both pathways. Preliminary in vivo study suggested that IL-36 has the potential for clinical application.

## 5. Conclusions

In conclusion, here, we propose a working model of IL-36 and IL-38 regulating FLS function via autophagy, which connects newly discovered IL-36/IL-38 immune regulation with autophagy, providing the possibility of clinical application targeting both pathways.

## Data Availability

The data used to support the findings of this study are available from the corresponding author upon request.
